# Completion of Vol. XVIII and the Future of the Journal

**Published:** 1879-07

**Authors:** 


### Completion of Volume Xviii, and the future of the Journal.

With the July number closes Volume Eighteen of this JOURNAL, offering a very opportune occasion for subscribers to pay up arrears, and for new ones to commence subscriptions. We hope to hear from all our friends without further or more pressing invitation.

Arrangements are now being made to transfer the JOURNAL to abler and more vigorous hands, and if completed, we have no doubt will result in advantage to the JOURNAL and its patrons. Perhaps we are hardly justified in announcing the new Editorial Staff, but we can assure our friends that it is to be a strong one and in full sympathy with the present friends of the JOURNAL. In taking leave of the JOURNAL and its kind friends, we reluctantly say farewell and God bless our successors, the JOURNAL and all its friends.

